# Paroxysmal Bruising in the Finger -Achenbach’s Syndrome-

**DOI:** 10.31662/jmaj.2018-0033

**Published:** 2018-11-12

**Authors:** Shin Watanabe, Naoyuki Hashiguchi, Hiroyuki Kobayashi

**Affiliations:** 1 Department of Emergency and Disaster Medicine, Juntendo University, Tokyo, Japan.; 2 Department of General Medicine, Juntendo University, Tokyo, Japan.

**Keywords:** Achenbach’s syndrome, Bruising, Finger

A 70-year-old man, who was not on anticoagulants, presented with sudden onset of spontaneous painless bruising of his left middle finger without prior trauma (Figure 1). After ruling out thrombocyte abnormalities, coagulopathy, and inflammation, we diagnosed Achenbach’s syndrome (AS). In 1958, Achenbach first described “paroxysmal hand hematoma” or “finger apoplexia” ^(1)^.

The cause of this disease is not known till date; however, pain and numbness develop suddenly followed by a hematoma at the same site. AS is more common in middle-aged women. The index and middle fingers are the most affected, and AS may be palmar ^(2)^. This disease does not spread to the fingertips, and AS is distinguishable from ischemic lesions. Specific treatment is not required, and the patient may recover spontaneously within 1 week with local rest. A general practitioner familiar with the disease can reassure the patient ^(3)^.

Informed written consent was obtained from the patient.

**Figure 1.**
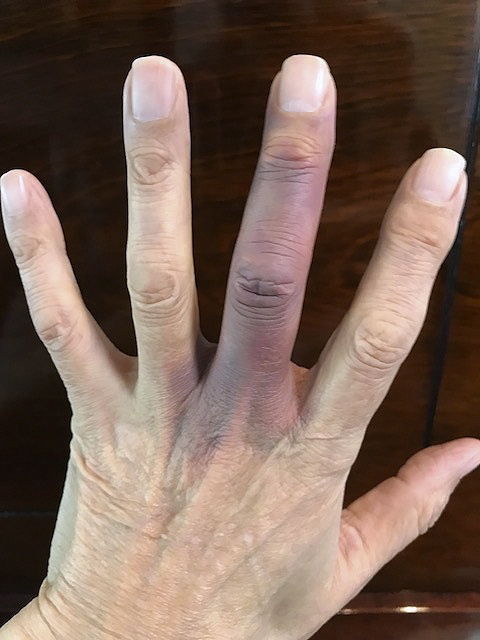
Bruising of the left middle finger.

## Article Information

### Conflicts of Interest

None

### IRB Approval Code

JHS 18-018 Juntendo University Hospital Independent Ethics Committee.
